# Baidu Index and COVID-19 Epidemic Forecast: Evidence From China

**DOI:** 10.3389/fpubh.2021.685141

**Published:** 2021-05-05

**Authors:** Jianchun Fang, Xinyi Zhang, Yang Tong, Yuxin Xia, Hui Liu, Keke Wu

**Affiliations:** ^1^School of Economics, Zhejiang University of Technology, Hangzhou, China; ^2^School of Accounting, Capital University of Economics and Business, Beijing, China; ^3^School of Economics and Management, Zhejiang Sci-Tech University, Hangzhou, China; ^4^School of Economics and Management, Southwest Jiaotong University, Chengdu, China

**Keywords:** Baidu index, coronavirus epidemic, N95 masks, Wuhan epidemic, forecast

## Abstract

With the global spread of the Coronavirus epidemic, search engine data can be a practical tool for decision-makers to understand the epidemic's trends. This article uses trend analysis data from the Baidu search engine, the most widely used in China, to analyze the public's attention to the epidemic and the demand for N95 masks and other anti-epidemic materials and information. This kind of analysis has become an important part of information epidemiology. We have analyzed the use of the keywords “Coronavirus epidemic,” “N95 mask,” and “Wuhan epidemic” to judge whether the introduction of real-time search data has improved the efficiency of the Coronavirus epidemic prediction model. In general, the introduction of the Baidu index, whether in-sample or out-of-sample, significantly improves the prediction efficiency of the model.

## Introduction

In recent years, with the rapid development of mobile networks, web search data has been widely used in epidemiological research. Internet search data not only helps governments and medical institutions to make market forecasts quickly and effectively, but the Internet has also become an important platform for the government to issue epidemic and stay-at-home orders. The general public has gradually formed the habit of searching for epidemic information, treatment methods, and drug purchases using the Internet. As a result, Internet search data has gradually become a new and time-sensitive data source for studying epidemic trends and public opinion. Starting with the SARS epidemic in China in 2003, the continued existence of MERS-CoV in the Middle East since 2012, and the Coronavirus epidemic from 2019 to the present, the public's attention to epidemics has increased. Determining how to make use of the public's attention as a reference point for government and medical system decision-making is becoming the focus of information epidemiology.

During the COVID-19 pandemic, most countries adopted stay-at-home policies in order to prevent the virus' spread. This led to a decrease in the number of patients in some outpatient clinics. However, their diseases were hidden or postponed after the COVID-19 pandemic. Home isolation policies further promoted the widespread use of search engines and have had a profound impact on the lifestyle of the general public. People are increasingly seeking solutions through the Internet, including searching for information about health problems, symptoms, and treatment options. Especially in the midst of the epidemic, information on COVID-19 treatment options and drugs is crucial to safeguarding public health. It can be expected that during the current COVID-19 pandemic, the number of people using the Internet to retrieve health information will greatly increase. Therefore, a survey of public trends using search engine data may provide clues about the frequency of certain diseases during the COVID-19 epidemic. However, epidemic research using Chinese Internet search data has just started.

Due to the fact that China cannot use Google search services, most people use Baidu as an information search tool. Baidu had a market share of 78.4% in China in February 2021. Thus, disease keywords frequently searched on the Baidu search engine can provide information about the disease spectrum of the population. Data from Baidu can provide timely and effective information transmission channels for epidemic monitoring, emergency response, and public opinion guidance. Many studies have used Google Trends to measure interest in infectious diseases and public awareness of diseases. However, it is uncommon to apply the Baidu index to research in emerging information epidemiology. Therefore, we use the Baidu index as a data analysis tool to establish a theoretical analysis model to compare the model containing the Baidu index and the benchmark model. This analysis provides a reference for the formulation of epidemic policies by relevant decision-making agencies.

Research on COVID-19 has been extensive. And most of the existing literature has been concentrated on those disease-related Google Trends keywords that were searched during the epidemic, namely skin diseases, smoking cessation, hand washing, and smell (1). Some studies consider epidemic trend forecasts (2–4). Sousa-Pinto et al. (5) investigates the change in the purpose that Google Trends has been used for. In recent years, the purpose of Google Trends has shifted from public opinion monitoring to trend predicting (6, 7). There are also some studies that argue that monetary policy and stock market may respond to the news contained in the Baidu index and using the Baidu index to monitor and predict the trend in the number of confirmed cases in China (8–17).

## Data Source and Keyword Selection

Most Chinese Internet users use Baidu, a search engine similar to Google. Data such as the number and regional distribution of certain keywords searched by Chinese users will be reflected through the Baidu index. The Baidu index allows the collection of data about user searches. In this case, we can use Baidu trend data to judge or predict the epidemiological characteristics of China. The search language is Chinese.

The Baidu index roughly follows the principle proposed by (18). The data collected from the Baidu index is standardized, and researchers can select data from different geographic regions, across genders and other characteristics, from a custom sample time range.

To examine Chinese people's attention to the epidemic, we chose “Coronavirus epidemic,” “N95 mask,” and “Wuhan epidemic” as search keywords from December 31, 2019 to March 11, 2021. The peak for the keyword “Coronavirus epidemic” was January 23, 2020. The peaks of the keywords “N95 mask” and “Wuhan epidemic” both appeared on January 25, 2020. However, it is worth noting that searches using the keyword “N95 mask” on December 31, 2019, the day the Wuhan epidemic was declared, increased by six times the search volume for masks the day before. There was no second peak following the peaks for these keywords. This is different from other countries or regions where we see several peaks. This is because the Chinese government announced on January 23, 2020 that Wuhan would be closed down, which had an immediate effect on the control of the epidemic. The public's attention to the epidemic never returned to the level of the first peak.

An interesting fact is that the first new case appeared on January 21, 2021, which is 21 days after the significant increase in the use of the “N95 masks” search term according to the Baidu index. In other words, by analyzing the Baidu index search terms, signs of the epidemic can be detected 21 days in advance. Similarly, when new cases reached a short-term peak on August 13, 2020, Baidu index-related search terms such as “Coronavirus epidemic,” “N95 mask,” and “Wuhan epidemic” reached their peaks 16, 15, and 7 days earlier, respectively. Thus, search engine trend data can be used as leading indicators for epidemic monitoring, early warning, and preparation of medical supplies.

Because data on the keyword “Coronavirus epidemic” was not available until February 26, 2020, the data sample period for the empirical part of this study is from February 26, 2020 to March 11, 2021.

From the Baidu index, we can also see a significant trend: Chinese people pay more attention to the epidemic in their own country. The global epidemic has no significant impact on the number of searches. For example, on February 11, 2020, the World Health Organization announced the official name of the epidemic, COVID-19. However, the search index did not rebound significantly after this official announcement. At that time, the epidemic in China had been significantly controlled. This is significantly different from epidemic trends in other countries and regions. Data on new cases in China comes from the National Health Commission, and the data interval covers February 26, 2020 to March 11, 2021.

## Statistical Description and Empirical Research

Given that Baidu is the most popular Internet search engine in China. This article uses Baidu index data to reflect the public's focus on the epidemic, and applies it to the forecast of epidemic trends. A higher number means more users search for the keywords within the set location and time period. The Baidu index is a real-time example of Baidu search data, which has been available since January 1, 2011. Downloading data from the Baidu index is a paid service, whereas Google Trends data is available for free. We use the Baidu index to measure the degree to which Chinese people are paying attention to COVID-19 and to improve capacity to predict new COVID-19 cases.

During the period of our study, the highet number of new cases of COVID-19 was 3,622 (Table 1), occurring on February 27, 2020, and there has generally been on a downward trend since then. The minimum value is 0, obtained on June 21, 2020. The maximum value of search keywords for “Coronavirus epidemic” is 2,803, obtained on July 24, 2020. The minimum value is 448, obtained on March 5, 2021. This is mainly due to the fact that most of the recent epidemics in China have been imported from abroad, and the public's attention to the epidemic has dropped significantly. The maximum search term of “N95 mask” was 25,176, which was also obtained on February 27, 2020, shortly after the outbreak began. The minimum value was obtained on February 15, 2021. The maximum value of the keyword “Wuhan Epidemic” was obtained on February 26, 2020, which was the first day of the sample period. The data has been repeated since then; while all increased slightly, none exceeded their previous peak.

**Table 1:** Statistical description of the main variables.

	**COVID**	**Corona epidemic**	**N95 Mask**	**Wuhan epidemic**
Mean	148.5053	1197.234	1727.184	3274.85
Median	19	975	696	2,296
Maximum	3,622	2,803	25,176	20,712
Minimum	0	448	219	854
Std. Dev.	470.1087	464.9004	3378.473	2897.205
Skewness	4.641613	0.88965	4.938219	2.491931
Kurtosis	25.72972	2.959039	29.67639	10.29989
Jarque-Bera	9544.629	50.15347	12811.92	1237.015
Probability	0	0	0	0
Sum	56,432	454,949	656,330	1,244,443
Sum Sq. Dev.	83759843	81,914,168	4.33E+09	3.18E+09
Observations	380	380	380	380

We use an autoregressive model to check the relationship between new COVID-19 cases and the Baidu index. The model is as follows:

(1) $$\begin{array}{r} Y_{t}=\sigma +\overset{n}{\underset{i=1}{\sum }}\beta_{i}Y_{t-i}+\varepsilon_{t} \end{array}$$

To examine the impact of the Baidu index of different keywords on the new cases of COVID-19, we added the Baidu index to the model. The extended model is as follows:

(2) $$\begin{array}{r} Y_{t}=\sigma +\overset{n}{\underset{i=1}{\sum }}\beta_{i}Y_{t-i}+\theta X_{t}+\varepsilon_{t} \end{array}$$

Among them, *Y*_*t*_ represents the number of new COVID-19 cases in China every day, *Y*_*t*−*i*_ is a lagging item, and C represents the Baidu index level of keywords such as “Coronavirus epidemic,” “N95 mask,” and “Wuhan epidemic.” σ, β_*i*_, and θ are parameters. ε_*i*_ is the error term.

The calculation results in Table 2 show that, except for the coefficient of the “Coronavirus epidemic” keyword in Model 1, which failed the significance test, the other models passed the 1% significance test. The calculation results of Model 4 show that the greater the number of people who used the search term “N95 masks,” the more serious the epidemic and the stronger the enthusiasm of people to buy N95 masks to enhance their self-protection. Similarly, the greater the number of people using the search term “Wuhan epidemic,” the more severe the epidemic. People want to know more about the progress of the epidemic. The number of searches for “N95 mask” and “Wuhan epidemic” is directly proportional to the new cases of COVID-19. However, the coefficient of the search term “Coronavirus epidemic” is negative. This is because searching for “Coronavirus epidemic” indicates that people feel more concerned about the severity of the epidemic and their internet searches enhance their awareness of protection measures, helping to control the spread of the epidemic. The coefficients of Model 2 and Model 3 are also positive, and the value of the coefficients does not change much, which shows that the model has a certain robustness.

**Table 2:** Forecast results of different models.

	**Model (0)**	**Model (1)**	**Model (2)**	**Model (3)**	**Model (4)**
COVID_t−1_	0.944^***^	0.943^***^	0.795^***^	0.914^***^	0.752^***^
Coronavirus epidemic		0.003			−0.043^***^
N95 Mask			0.023^***^		0.027^***^
Wuhan epidemic				0.006^***^	0.008^***^
Obs.	379	379	379	379	379
S.E.	79.322	79.416	75.649	78.663	74.411
Adj. R^2^	0.969	0.969	0.972	0.970	0.973
F	11856.65	5914.385	6537.191	6031.729	3381.952

*** *Significance level of 1%*.

**Figure 1:**
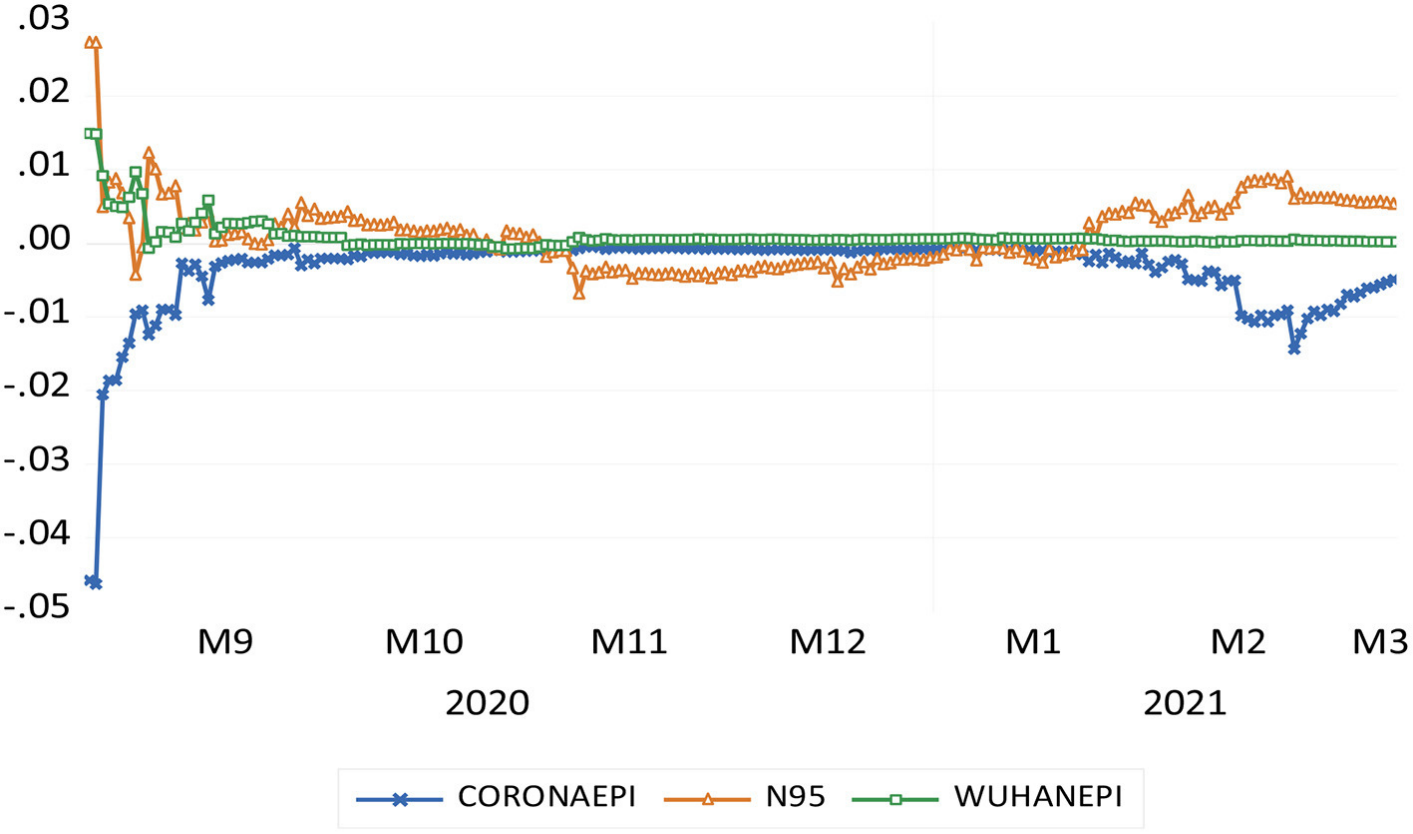
Time-varying parameter trends of independent variables.

Although most of the above three keywords passed the significance test, we still need to examine the stability of the coefficients of equation (2) during the rolling regression period of the sub-sample. To examine this result, we compressed the data sample from February 26, 2020 to August 25, 2020. Model 2 is used as the regression base of half a year for rolling estimation until the end of the sample period. In other words, the second regression is estimated to be from February 27, 2014 to August 26, 2020, and so on. The calculation results are shown in Figure 1.

In the process, parameter θ of the independent variable is extracted. The value of the θ parameter is mainly used to investigate the stability of the relationship between the independent variable and the dependent variable. The ideal situation is that the parameters do not change significantly during the sample period. Figure 1 shows the evolution trend of the coefficients of the three Baidu indexes.

First, the coefficient of the “Wuhan Epidemic” keyword is relatively stable. Except for the large change in the coefficient at the beginning of the sample period, the coefficients thereafter fluctuate at around 0.1, and the fluctuation range gradually narrows.

Previously, the coefficient of “N95 masks” fluctuated greatly, and most were positive before November 2020. The coefficient was negative in November and December 2020 and gradually changed to positive after January 2021. This shows that people's retrieval of N95 mask keywords changes with the fluctuation of the epidemic, so the overall performance of the parameters is unstable.

The “Coronavirus epidemic” coefficient fluctuations are also relatively large. From September 21, 2020 to January 31, 2021, the coefficient is positive. However, in the rest of the period, the coefficient is negative and fluctuates greatly. This is related to the serious epidemic situation at the beginning of the sample and the repeated epidemic situation in some periods.

The Baidu index helps us to make early judgments about changes in the epidemic. If the forecast is accurate, it will be possible to gain insight into the epidemic's trend and the public's demand for information before official data is released. We use the one-step forward method to compare the prediction performance of the Baidu index expansion model and the benchmark model. This benchmark model is defined as a constant forecast, that is, the epidemic trend changes at the same rate as the previous observations. The new model is expanded on this basis: the one-step forward prediction on September 3, 2020 is based on the regression equation of Model 2, and the sample range is from February 26, 2020 to September 2, 2020. We use the root mean square prediction error (RMSFE) to evaluate the accuracy of the model's prediction. That is, we compare the improvement degree of the extended model relative to the root mean square prediction error of the benchmark model to evaluate the overall performance of the model.

Table 3 provides one-step forward prediction performance of various extended models relative to the benchmark model. On the whole, the extended model performs somewhat better than the prediction of the benchmark model. Among them, Model 4 performed best in one-step forward prediction, with an improvement of 24.7%. The improvement of Model 3 is small.

**Table 3:** Out-of-sample forecast performance.

	**Model (0)**	**Model (1)**	**Model (2)**	**Model (3)**	**Model (4)**
RMSFE	143.3993	109.9745	116.9652	138.9774	108.0304
RMSFE/BENCH	1.00	0.767	0.816	0.969	0.753
Percentage	n/a	23.3%	18.4%	3.1%	24.7%
improvement					
Theil inequality	0.148833	0.124738	0.126399	0.153554	0.116358
coefficient					

## Research Conclusions and Policy Recommendations

As the COVID-19 epidemic continues to spread around the world, the public's awareness of self-protection continues to increase. People are actively looking for official information about epidemics, drugs, and other concerns on the Internet. Therefore, in the context of a global pandemic, we use the Baidu index to monitor the trend of the epidemic in China in real time, and to detect symptoms that have not been identified so far. The Baidu index can even be used to identify those with mild or asymptomatic symptoms. These people may not receive the attention of traditional medical channels. Therefore, this trend judgment tool has extremely attractive prospects. This article uses the monitoring tool of the Baidu index to judge the peak of public attention to the epidemic's trends. This can not only help identify disease-related symptoms, but also further identify the affected population and improve the accuracy of epidemic forecasts.

Our findings show that since the public searches for health-related information on the Internet, web search queries are a valuable source of information about collective health trends. The Baidu index provides an effective experimental tool. We can monitor and analyze the behavior of seeking information on epidemics and medical care through the query forms of search engines to detect outbreaks in near real time. We use the Baidu index to test this in the context of the epidemic in China. Preliminary tests have shown that analyzing data from the Baidu index to judge epidemic trends can allow us to detect outbreaks of COVID-19 7–21 days earlier than official data. Relying on traditional laboratory and clinical data to publish weekly statistics for countries and regions usually results in a lag of 1–2 weeks. This is also consistent with our calculation results.

Judging from the calculation results of the time-varying parameter model of the three search terms—“Coronavirus epidemic,” “N95 mask,” and “Wuhan epidemic”—the coefficients of the keyword “Wuhan epidemic” is relatively stable, and the coefficients of “Coronavirus epidemic” and “N95” fluctuate greatly, closely related to the repeated epidemics in some periods. The extended model shows that the prediction improvement of Model 4 relative to the benchmark model reaches 24.7%.

Based on the above research, we believe that real-time monitoring and early warnings of outbreaks will help the public and health care professionals to formulate epidemic prevention and control measures in time, detect cases on time, and ensure that patients receive adequate treatment, thereby reducing incidence and death rates. Whether it is SARS, Middle East Respiratory Syndrome, or COVID-19, this research reminds the international community of the need for the real-time monitoring of new infectious diseases, bioterrorism, and pandemics. The big data generated by the epidemic is not only useful for public health practice, but also helps to improve the efficiency of clinical decision-making and research. Our research shows that experts in the medical field, through cross-field cooperation, can use the data processing methods of the Baidu Index to improve the efficiency of disease surveillance and build tools for infectious disease surveillance. In addition, with the increasing dependence of the general public on the Internet, big data monitoring of the collective wisdom of the population can track the trends of infectious diseases and epidemics faster than traditional monitoring systems. The use of these innovative technologies brings us closer to true real-time outbreak monitoring.

Finally, we need to develop novel ways to communicate with the public about infectious diseases like COVID-19. The use of digital tools not only helps policy makers understand the public interest, but also allows for tailored, targeted messages for the public, making it possible to improve the effectiveness of epidemic propaganda. The analysis of Baidu index and other data can complement the advantages of traditional public health monitoring systems, improve the emergency response capabilities of the medical care system, and improve the benefits for stakeholders and the level of medical care services. The Baidu index data might be used by government to monitor the trend of the epidemic. This has remarkable significance for promoting international cooperation in epidemic surveillance and the cross-border distribution of vaccines.

## Data Availability Statement

Publicly available datasets were analyzed in this study. This data can be found at: http://index.baidu.com.

## Author Contributions

JF: writing-original draft. XZ: policy suggestions. YT: literature. YX: investigation and software. HL: proofreading. KW: conceptualization and supervision. All authors contributed to the article and approved the submitted version.

## Conflict of Interest

The authors declare that the research was conducted in the absence of any commercial or financial relationships that could be construed as a potential conflict of interest.
